# Inhibitory Activity of YKL-40 in Mammary Epithelial Cell Differentiation and Polarization Induced by Lactogenic Hormones: A Role in Mammary Tissue Involution

**DOI:** 10.1371/journal.pone.0025819

**Published:** 2011-10-03

**Authors:** Steve Scully, Wei Yan, Brooke Bentley, Qing Jackie Cao, Rong Shao

**Affiliations:** 1 Pioneer Valley Life Sciences Institute, University of Massachusetts, Springfield, Massachusetts, United States of America; 2 Molecular and Cellular Biology Program, Morrill Science Center, University of Massachusetts, Amherst, Massachusetts, United States of America; 3 Department of Pathology, Baystate Medical Center, Tufts University, Springfield, Massachusetts, United States of America; 4 Department of Veterinary and Animal Sciences, University of Massachusetts, Amherst, Massachusetts, United States of America; International Centre for Genetic Engineering and Biotechnology, Italy

## Abstract

We previously reported that a secreted glycoprotein YKL-40 acts as an angiogenic factor to promote breast cancer angiogenesis. However, its functional role in normal mammary gland development is poorly understood. Here we investigated its biophysiological activity in mammary epithelial development and mammary tissue morphogenesis. YKL-40 was expressed exclusively by ductal epithelial cells of parous and non-parous mammary tissue, but was dramatically up-regulated at the beginning of involution. To mimic ductal development and explore activity of elevated YKL-40 during mammary tissue regression *in vivo*, we grew a mammary epithelial cell line 76N MECs in a 3-D Matrigel system in the presence of lactogenic hormones including prolactin, hydrocortisone, and insulin. Treatment of 76N MECs with recombinant YKL-40 significantly inhibited acinar formation, luminal polarization, and secretion. YKL-40 also suppressed expression of E-cadherin but increased MMP-9 and cell motility, the crucial mechanisms that mediate mammary tissue remodeling during involution. In addition, engineering of 76N MECs with YKL-40 gene to express ectopic YKL-40 recapitulated the same activities as recombinant YKL-40 in the inhibition of cell differentiation. These results suggest that YKL-40-mediated inhibition of cell differentiation and polarization in the presence of lactogenic hormones may represent its important function during mammary tissue involution. Identification of this biophysiological property will enhance our understanding of its pathologic role in the later stage of breast cancer that is developed from poorly differentiated and highly invasive cells.

## Introduction

Mammary gland development during childhood does little more than keep pace with the general growth of the body until puberty. During puberty, the mammary gland exhibits a substantially dynamic process through which it gives rise to highly organized ductal branches from earliest rudimentary ducts [1], [2]. The biophysiological property of these ductal branches is characterized by continuous proliferation, migration, and differentiation of ductal epithelial cells and their adjacent myoepithelial cells, thus creating epical-basal luminal buds also referred to as acini, a basic functional unit of the mammary gland [3]. During pregnancy and lactation, the mammary glands undergo vigorous proliferation and differentiation into a fully branched ductal network that orchestrates a secreted duct system capable of producing and collecting milk protein.

It is well established that proper organization, maintenance, and function of the mammary ducts are largely ascribed to cell-cell adhesion and polarization of ductal epithelium and its interaction with extracellular matrix (ECM) [4]. These epithelial cells coordinate together to generate and maintain a polarized cellular layer that is surrounded by myoepithelial cells and ECM, contrary to inner acinar cells that lack attachment to ECM and rapidly undergo apoptosis, a death program analogous to anoikis [5], [6]. The ECM-rich basement membrane is able to interact with epithelial cells through binding different integrins accumulated at the abluminal membrane and to induce activation of FAK, PI3K, and Bcl2; thus enhancing the epical-basal polarization and survival of epithelial cells [7], [8].

YKL-40, also known as human cartilage glycoprotein-39 or chitinase-3-like-1, is a secreted glycoprotein originally identified from culture medium of a human osteosarcoma cell line MG-63 [9]. Structural analyses of YKL-40 have demonstrated that YKL-40 is highly conserved in human [10], porcine [11], cow [12], mouse [13], rabbit [14], and goat [15]. Putative YKL-40-like proteins were also found in *Drosophila* [16], bacteria [17], and zebra fish [18]. Human YKL-40 protein contains an open reading frame of 383 amino acids with a molecular mass of 40 kDa and it falls into a member of chitinase or 18-glycosyl-hydrolase gene family which can bind oligosaccharide. But it does not have chitinase/hydrolase activity because of the substitution of an essential glutamic acid with leucine in the chitinase-3-like catalytic domain [19], [20]. YKL-40 is normally expressed by a number of different cell types that include chondrocytes [21], synoviocytes [22], vascular smooth muscle cells [11], macrophages [23], and neutrophils [17]. However, its normal function in these cells is incompletely understood. In a broad spectrum of human inflammatory diseases, serum levels of YKL-40 are elevated, including bacterial infections [24], rheumatoid arthritis, osteoarthritis [25], hepatic fibrosis [26], and bowel lesion [27]. Thus the pathologic function of YKL-40 is implicated in the tissue remodeling and macrophage differentiation. Recently, YKL-40 null mice have exhibited markedly diminished antigen-induced Th2 inflammation and impaired macrophage activation and differentiation [28].

Over the past decade, multiple independent studies have demonstrated that high serum levels of YKL-40 are associated with metastasis and reduced survival in a variety of human carcinomas such as breast cancer [29], colorectal cancer [30], ovarian cancer [31], leukemia [32], and glioblastoma [33]. Consistent with these data, our recent reports unveiled an angiogenic signature of YKL-40 in the development of breast cancer and brain tumor [34], [35]. Although membrane receptors specific for YKL-40 binding have not yet been identified, the heparin-binding affinity of YKL-40 appears to be essential for its activity, resembling the heparin-binding property of other proteins such as extracellular matrix protein vitronectin and angiogenic factors bFGF and VEGF [34], [36], [37]. Furthermore, YKL-40 was able to induce focal adhesion kinase (FAK) and MAP kinase Erk1/2 signaling cascades that mediate cell adhesion, spreading, survival, and migration in vascular endothelial cells [34]. Likewise, YKL-40 displayed the ability to trigger phophoinositide 3-kinase (PI-3K)/AKT and MAPK signaling that regulates mitogenesis and survival of fibroblastic cells [38].

While the expression levels of YKL-40 in normal mammary tissue remain to be investigated, there is compelling evidence showing that YKL-40 levels are rapidly increased in the initiation of mammary tissue involution as compared to the levels during pregnancy and lactation [12], [15]. For example, oligonucleotide microarray data analyzing a pregnancy-involution cycle of the mammary tissue demonstrated that YKL-40 was ranked as one of the top candidates in 145 up-regulated genes specific for involution [39], [40]. Consistent with these gene microarray data, YKL-40 protein levels were also detectable in milk secretion from weaning gland of bovine and goat, but were not detectable from lactating mammary tissue [12], [15], [41]. These data suggest that elevated YKL-40 may be associated with mammary gland regression. To establish a functional role for YKL-40 in the normal mammary gland morphogenesis, here we tested the hypothesis that YKL-40 inhibits mammary epithelial differentiation and development during mammary gland development.

## Results

**Figure 1:**
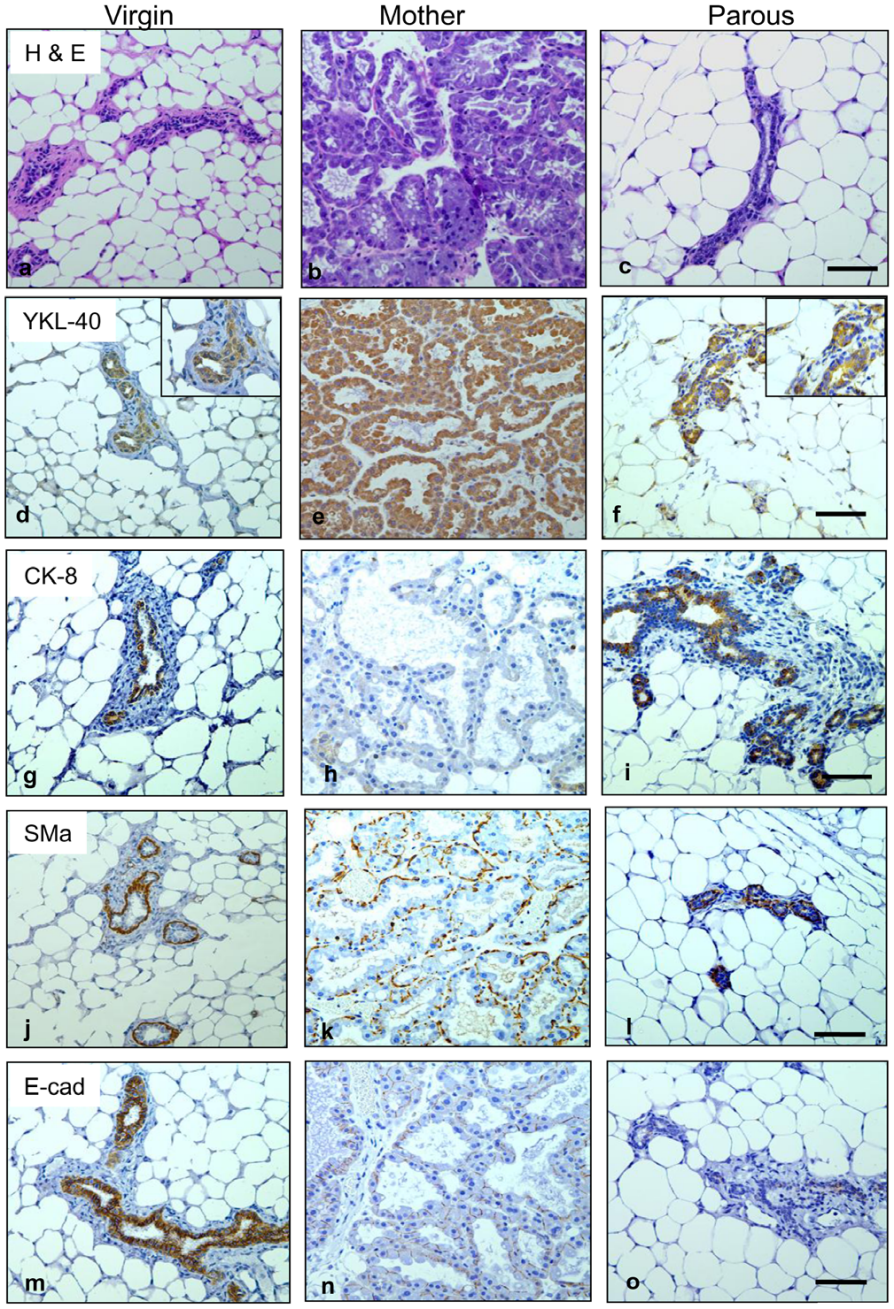
Expression of YKL-40 is highly increased in mammary epithelial cells at the beginning of involution when epithelial markers diminish. Mammary tissue specimens removed from virgin mice, mothers at the initiation of involution, and parous mice were analyzed for H & E staining (a-c), IHC of YKL-40 (d-f), CK-8 (g-i), SMa (j-l), and E-cad (m-o). Inserts in d and f represent a high magnification. Bars: 200 µm.

To investigate expression levels of YKL-40 during mammary tissue development, we utilized an immunohistochemical (IHC) approach in a series of different ages of parous and non-parous mice, including virgin mice (3-month old), mothers at the beginning of involution (7-month old), and parous animals (10-month old). Hematoxylin and eosin (H & E) analysis revealed that a few mammary ducts were scattered in fat-rich stroma from both virgin mice and parous mice, whereas extensive secreted ducts were observed in the tissue from early involution (Figure 1a-c). In IHC staining, expression levels of YKL-40 were detectable exclusively in ductal epithelial cells but not in others (*e.g.* myoepithelial cells) in virgin mice. However, its levels were noticeably evaluated in the ductal epithelial cells from weaning tissue. After involution, the remaining ducts markedly decreased expression of YKL-40 (Figure 1d-f), suggestive of its function associated with ductal regression. The specificity of this anti-YKL-40 antibody (rAY) was validated by a test that pre-incubation of recombinant YKL-40 with rAY protected the interaction between tissue-derived YKL-40 and rAY, whereas collagen IV failed to resemble the inhibition of recombinant YKL-40 (Figure S1). In addition, pre-immune serum rabbit IgG did not recognize YKL-40 in the tissue. Staining with an epithelial marker cytokeratin-8 (CK-8) showed that ductal epithelial cells of the virgin mammary tissue expressed CK-8, but its level was significantly reduced during involution. In the parous mice, CK-8 was resumed to the basal levels expressed in the virgin animals (Figure 1g-i). In contrast, a myoepithelial marker smooth muscle alpha actin (SMa) remained at a strong expression level in the tissue from all different ages (Figure 1j-l), indicating that myoepithelial cells do not play a major role in the mammary gland development. Interestingly, an epithelial surface marker E-cadherin (E-cad) responsible for cell-cell adhesion was significantly decreased during and after weaning compared with the ducts from virgin mice (Figure 1m-o). These data suggest that ductal epithelial property is lost during involution and this physiological change may be associated with elevated expression of YKL-40.

**Figure 2:**
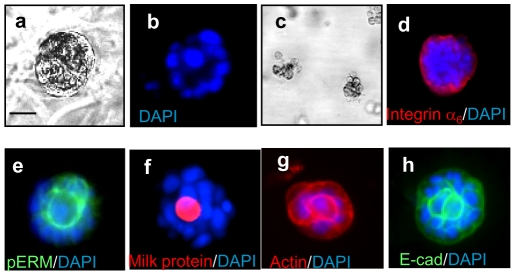
Differentiation and polarization of 76N cells into alveolus-like structure in 3-D Matrigel in the presence of PRL, HDC, and INS. Normal mammary epithelial cells 76N MECs were suspended with RPMI assay medium containing 4% Matrigel in the presence (a,b,d, e, f, g, & h) or absence (c) of 5 µg/ml PRL, 5 µg/ml HDC and 5 µg/ml INS for 2 weeks. Acinus-like structure was visualized under a microscope (a). Acinar polarization was analyzed by immunofluorescent staining with single nuclear staining of DAPI depicting a hollow lumen (b), and double staining using DAPI and anti-integrin α6 (d), anti-pERM (e), anti-milk protein (f), anti-actin (g), or E-cad (h) antibody. Bars: 50 µm.

This *in vivo* data has encouraged us to test the likelihood that increased expression of YKL-40 inhibits differentiation of mammary epithelial cells. To explore this, we employed an *in vitro* three dimensional (3-D) matrix culture system in the presence of lactogenic hormones including prolactin (PRL), hydrocortisone (HDC) and insulin (INS), which represents the most common *in vitro* model capable of mimicking acinus-like secretion, differentiation, and polarization of ductal epithelial cells *in vivo* [42], [43]. A normal human mammary epithelial cell line 76N MECs were employed for the study. 76N MECs were initially established through transduction of a gene encoding human telomerase catalytic protein [44], [45], [46] and they possessed mammary stem cell/progenitor cell properties with ability to self-renew and differentiate into luminal epithelial and myoepithelial cells [46]. 76N MECs were plated in growth factor-reduced Matrigel supplemented with PRL, HDC, and INS over 14 days. These cells aggregated to form an outer layer of a sphere, typically creating a lumen in the center. This phenotype demonstrated an apical-basal polarization of ductal epithelial cells (Figure 2a & 2b). In contrast, lack of PRL, HDC and INS (Figure 2c) failed to stimulate this acinar polarization. In order to validate the differentiation and polarization capacity of 76N MECs, we tested for both a basal cell marker integrin α6 and an apical cell marker phosphoEzrin/Radixin/Moesin (pERM). As shown in Figure 2d & 2e, the sphere demonstrated the polarization with strong staining of integrin α6 on the basal side and pERM on the apical surface. Secreted milk protein was accumulated in the lumen (Figure 2f). In addition, cytoskeleton protein actin and E-cad were also stained by the spheres (Figure 2g & 2h). Altogether, these results suggest that 76N MECs retain differentiation properties of mammary epithelial cells capable of orchestrating a secreted luminal epithelium *in vitro*.

**Figure 3:**
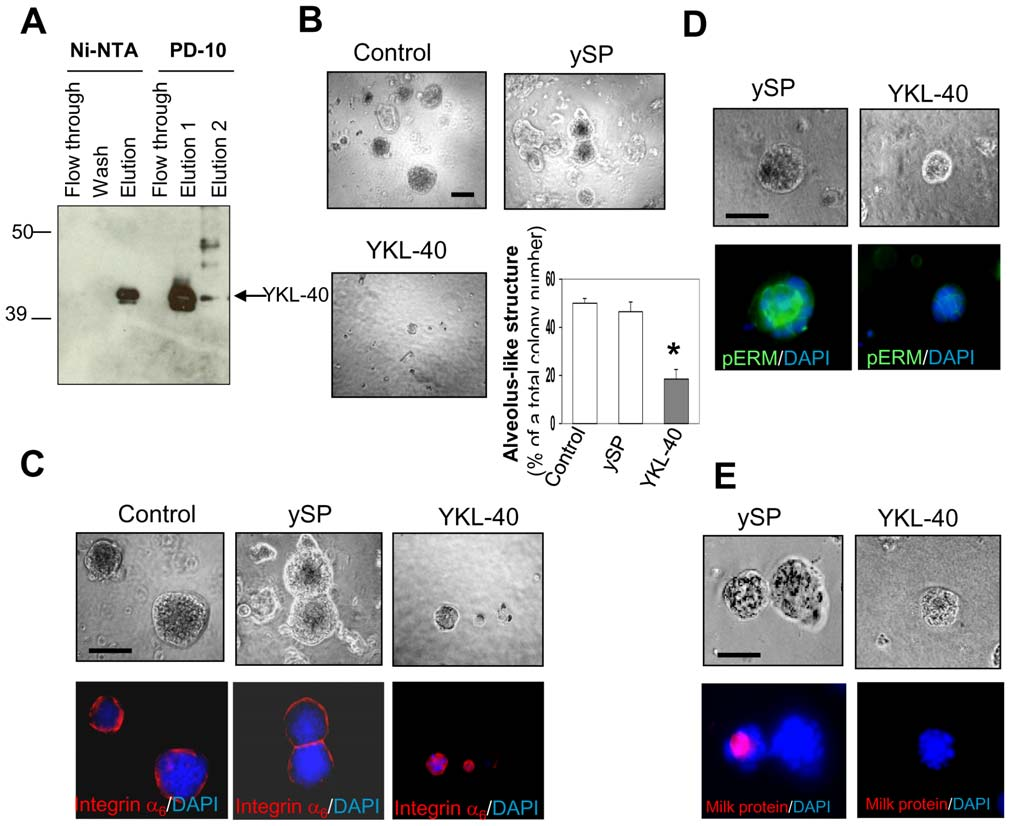
YKL-40 inhibits differentiation, polarization, and milk secretion of 76N MECs in 3-D Matrigel. Recombinant YKL-40 collected from baculoviral medium was applied to an affinity his-binding Ni-NTA column. The final recombinant YKL-40 was purified through a desalt PD-10 column. Samples were analyzed by immunoblotting using anti-YKL-40 polyclonal antibody (A). 76N MECs were grown in the assay medium described in Figure 2 in the presence of 100 ng/ml ySP or YKL-40 for 2 weeks. Formation of acinus-like structure (diameter larger than 50 µm) in total colonies was quantified from each well (B). n = 5. *P<0.05 compared with control or ySP. The alveolar structure was subjected to immunofluorescent staining using DAPI and anti-integrin α6 antibody (C), anti-pERM (D), and anti-milk protein (E) antibody. Bars: 50 µm.

Next, we treated these cells in the 3-D system with recombinant protein YKL-40 that was isolated and purified through a baculoviral infection system (Figure 3A). YKL-40 was found to inhibit the acinar structure in 14-day culture by 60% of the controls in the absence or presence of YKL-40 small peptide (ySP) that contains 20 amino acids of the C-terminus of YKL-40 (Figure 3B). Immunocytochemical analyses of acinus-like structures unveiled that YKL-40 dramatically restrained the alveolus formation and growth, as pERM and integrin α6 did not correspondingly localize to an apical and basal side in the presence of YKL-40, in contrast to control cells which exhibited a larger, polarized acinus and produce milk protein in the lumen (Figure 3C-3E). YKL-40 did not influence 76N MEC proliferation as compared to the control cells or the cells treated with YSP in a monolayer culture dish (Figure S2). These data suggest that YKL-40 displays the ability to block 76N MEC secretion, differentiation, and polarization in the presence of lactogenic hormones.

**Figure 4:**
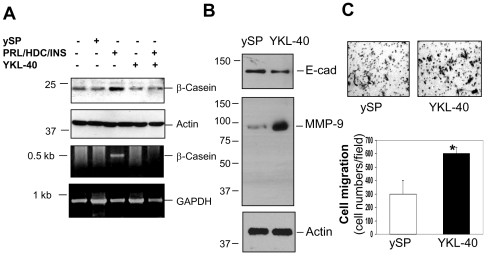
YKL-40 inhibits expression of β-casein and E-cadherin; but increases MMP-9 expression and cell migration. **A**. 76N MECs were grown to sub-confluence and added with 100 ng/ml ySP, 100 ng/ml YKL-40 or 5 µg/ml combined PRL, HDC and INS for 2 days. Cellular protein and RNA samples were collected to test expression of β-casein by immunoblotting and RT-PCR analysis, respectively. **B**. Two days following treatment as above, cell conditioned medium and cell lysates were subjected to immunoblotting using antibodies against E-cad and MMP-9. **C**. Cells were also determined for cell motility using a migration assay described in the Method and data were quantified. *P<0.05 compared with ySP control. n = 4.

In an attempt to further determine the regulation of epithelial secretion by YKL-40, we measured expression of a milk protein β-casein in 76N MECs by immunoblotting and RT-PCR analysis. Consistent with the early immunocytochemistry results, β-casein was upregulated at both transcriptional and translational levels in response to PRL, HDC, and INS (Figure 4A). However, treatment with YKL-40 abolished the induction of β-casein, confirming its inhibitory impact on epithelial secretion. Next, to address if YKL-40 has the ability to alter expression of E-cad and MMP-9, both of which play an important role in the cell polarity, motility, and extracellular matrix remodeling, the critical mechanisms that mediate mammary gland regression during involution. YKL-40 noticeably inhibited expression of E-cad but increased MMP-9 relative to controls treated with ySP (Figure 4B). Accordingly, we evaluated cell motility using a cell migration assay. YKL-40 increased cell migration approximately 2-fold greater than did ySP (Figure 4C). Collectively, these data suggest that treatment of mammary epithelial cells with YKL-40 leads to inhibition of cell secretion, differentiation and polarization, and increases in cell motility.

**Figure 5:**
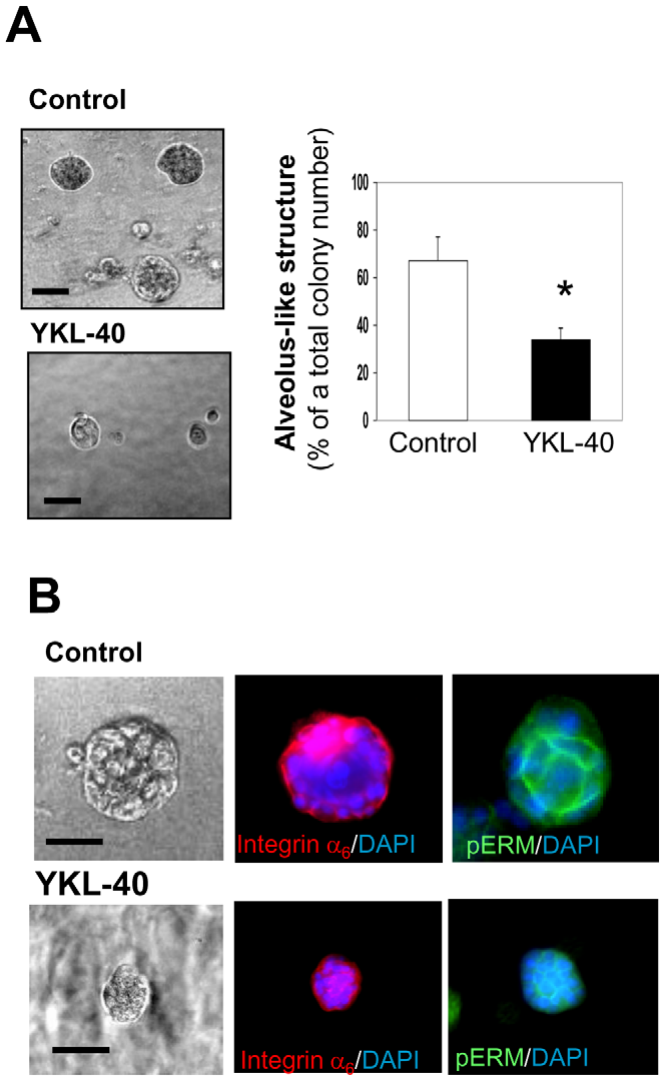
Ectopic expression of YKL-40 in 76N MECs results in inhibition of cell differentiation and polarization in 3-D Matrigel. **A**. 76N MECs expressing ectopic YKL-40 or vector were employed for the study of cell differentiation in Matrigel as described in Figure 2. The acinus-like structure was quantitatively analyzed. n = 4. *P<0.05 compared with a control group. **B**. Polarization of these cells in Matrigel was co-stained with anti-pERM, anti-integrin α6 antibody, and DAPI. Bars: 50 µm.

To further validate this inhibitory signature for YKL-40 in the cells, we next determined if genetically acquired expression of YKL-40 can resemble the functions acted by recombinant YKL-40. Given that endogenous levels of YKL-40 in 76N MECs were not detectable (data not shown), we engineered a full length YKL-40 cDNA into the cells to express ectopic YKL-40. These cells resembled the activities of the parental 76N MECs exposed to recombinant YKL-40 in the Matrigel as studied earlier. Compared to vector control cells, 76N MECs expressing YKL-40 formed smaller acinus-like spheres (Figure 5A), as a total of spheres were reduced by 45%. Consistent with this inhibitory phenotype, 76N MECs expressing YKL-40 also demonstrated disruption of the apical-basal polarity, when the spheres were stained with pERM and integrin α6 (Figure 5B). The results strengthen our hypothesis that over-expression of YKL-40 inhibits epithelial cell differentiation and polarity.

**Figure 6:**
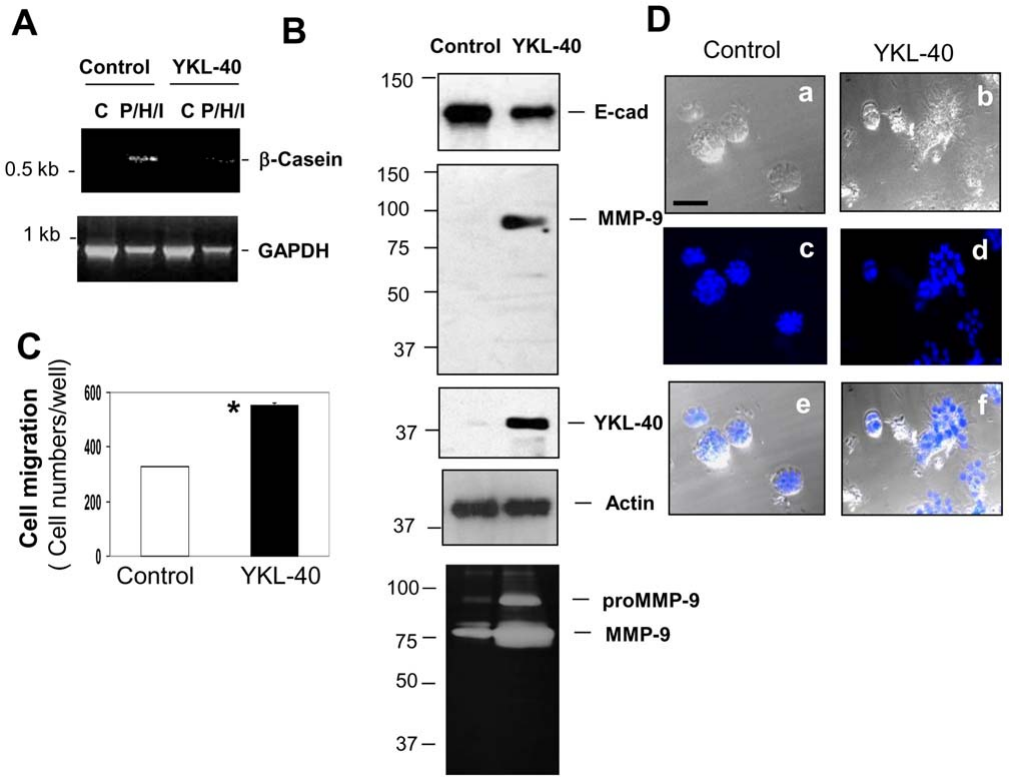
Over-expression of YKL-40 in 76N MECs decreases E-cad and β-casein, but increases MMP-9 expression and cell invasiveness. **A**. Cells ectopically expressing YKL-40 or vector were treated with the same condition as that used in Figure 4A. Cellular expression of β-casein was determined by RT-PCR as described in the Method. P: PRL; H: HDC; and I: INS. **B**. Cells over-expressing YKL-40 or vector were analyzed for expression of E-cad, MMP-9, and YKL-40 by immunoblotting and zymography. **C**. YKL-40-producing or control cells were tested for cell migration as migrated cells were quantified. n = 6. *P<0.05 compared with control cells. **D**. Cells over-expressing YKL-40 or vector were suspended with RPMI assay medium containing 2% Matrigel, 5 µg/ml PRL, 5 µg/ml HDC, and 5 µg/ml INS. After 7-day incubation, cells were fixed with 2% para-formaldehyde. Then nuclei were stained with DAPI and the cell morphology was analyzed with a single phase contrast (a, b), fluorescence (blue, c, d), and overlapping image (e, f). Bars: 50 µm.

We next sought to identify the impacts of YKL-40 on the expression of β-casein, E-cad, MMP-9, and corresponding invasive activity in these cells. Consistent with recombinant YKL-40 activity, ectopic expression of YKL-40 by 76N MECs resulted in suppression of β-casein and E-cad, but induction of MMP-9 and its activity (Figure 6A & 6B). Accordingly, YKL-40-expressing 76N MECs also demonstrated increased motility as cell migration was elevated by 72% relative to the control (Figure 6C). To further evaluate the invasive behavior acquired by the expression of YKL-40, we plated these cells on a diluted Matrigel that allows motile cells to spread and migrate. Following 7-day culture, 76N MECs expressing YKL-40 migrated and invaded the Matrigel, the phenotype contrary to the control cells that were restricted to grow as spheres (Figure 6D). These data are highly in line with our earlier findings employing recombinant YKL-40, demonstrating that YKL-40 plays a key role in the inhibition of epithelial cell differentiation and the increase of cell motility *in vitro*. Furthermore, these findings supported the notion from *in vivo* studies that strong induction of YKL-40 in the early involution is associated with impaired mammary epithelial property and subsequent mammary gland regression.

We also transplanted 76N MECs expressing YKL-40 into pre-cleared fat-pad tissue of SCID/Beige mice to monitor whether or not over-expression of YKL-40 has pathologic effects on normal mammary duct development. Mammary tissue developed from 76N MECs expressing ectopic YKL-40 exhibited well-organized epithelial ducts surrounded by fat-rich stroma, the phenotype identical to that found in mammary tissue derived from 76N MECs expressing an empty vector or host native cells in different ages of mice including virgin mice, mothers at the initiation of involution, parous and non-parous animals in H & E (data not shown) and IHC analysis of YKL-40 (Figure S3). Although higher levels of YKL-40 were expressed by ductal epithelial cells in mammary tissue bearing YKL-40-expressing 76N MECs than that in counterparts or host mammary tissue in the different ages of mice except the involution period, none of pathogenic events was observed. In addition, IHC analyses for ductal differentiation and other activities in these ages did not show significant difference between mammary tissues containing YKL-40-producing 76N MECs, control 76N MECs, or host native cells, including: CK-8, E-cad, SMa, estrogen receptor (ER), progesterone receptor (PR), cell proliferation marker Ki67, and cell apoptosis staining TUNEL (data not shown). The data suggest that YKL-40 over-expression in normal mammary epithelial cells does not have the ability to induce ductal pathogenesis.

## Discussion

Our current study has utilized multidisciplinary approaches including genetic engineering, 3-D Matrigel cultures, and cleared fat-pad xenotransplantation to characterize a functional role for YKL-40 in the normal mammary gland development. To our knowledge, this is the first time characterizing YKL-40 in the mammary tissue morphogenesis, which will enhance our understanding of biological and physiological properties of YKL-40 in this field.

We have found that YKL-40 is expressed merely by ductal epithelial cells in normal breast tissue at a low level throughout the life time except involution. This evidence suggests that the low expression of YKL-40 may be sufficient for the development of a limited numbers of mammary ducts in non-pregnant mammary tissue. In weaning, YKL-40 is markedly up-regulated in ductal epithelial cells, suggesting that YKL-40 may mediate mammary epithelial remodeling and regression. Consistent with our findings, milk levels of YKL-40 in goat and bovine were increasingly detectable during weaning; but were not detectable in lactation [12], [15], [41]. Furthermore, in the mimicking of an *in vivo* environment for ductal morphogenesis, we used a 3-D *in vitro* culture system in the presence of lactogenic hormones and found inhibitory effects of YKL-40 on epithelial cell differentiation, secretion, and polarization, highlighting a physiological role of YKL-40 in the inhibition of mammary duct differentiation during involution. It would be quite interesting to understand molecular mechanisms of how YKL-40 suppresses epithelial cell secretion and differentiation induced by lactogenic hormones.

During mammary gland development, epithelial morphogenesis and lateral ductal branching are controlled by a wide array of intra- and extra-cellular cues that are initiated from a variety of factors including: steroid hormones (*e.g.* estrogen and progesterone), polypeptide hormones (*e.g.* PRL, placental lactogenes), and growth factors (*e.g.* EGF, FGF, TGF-β, and insulin), ECM (*e.g.* laminin), and their binding receptors (*e.g.* integrins) [1], [3], [47], [48]. In addition, a cell death process anoikis also plays an active role in the polarization of luminal epithelium, the event that normally contributes to the establishment of a hollow ductal phenotype due to deprivation of ECM in the core of acini [5]. Aberrant expression or dysfunction of these factors could result in disruption of epithelial differentiation and acinar outgrowth, thus mediating or leading to a pathogenesis associated with mammary tumorigenesis [49], [50], [51]. However, over-expression of YKL-40 alone by epithelial cells in the current study does not initiate pathogenesis towards epithelial dysplasia, hyperplasia, or carcinogenesis. These data suggest that YKL-40 is not tumorigenic. But, it may collaborate with other oncogenic factors in participation of tumorigenesis, as YKL-40-mediated inflammation is implicated in the development of ulcerative colitis-associated neoplasia in colorectal tissue [52], [53]. It is well established that elevated YKL-40 is associated with the malignancy of breast cancer. For example, there is accumulating evidence indicating that elevated serum levels of YKL-40 correlate with breast cancer progression and decreased disease-free survival [29], [54]. We recently identified that cancer tissue expression of YKL-40 was associated with tumor vasculature formation, demonstrating the angiogenic property for YKL-40 during breast cancer development [34]. In context with current findings, all the data indicate that YKL-40 plays a pathological role in the late stages of breast cancer, rather than in the initial phase of the cancer.

Normal mammary epithelial cells express strong E-cadherin, a membrane protein characteristic of cell-cell tight contacts. Dysfunction of E-cadherin is associated with loss of intercellular adhesion, the process of which is fundamental during mammary gland involution [55]. For example, truncated E-cadherin in mammary epithelial cells led to cell-cell disassociation and thus loss of ductal epithelial polarization [55]. In addition, increased expression of MMPs in mouse mammary epithelial cells was correlated with decreased E-cadherin, both of which mediate disruption of cell polarity and contribute to invasiveness [56]. Our current study found that YKL-40 inhibited E-cadherin and induced MMP-9 expression in 76N MECs, concurrent with the changes in cell polarity and invasiveness in 3-D Matrigel culture, thus demonstrating an in important role played by YKL-40 during mammary tissue remodeling and involution. We also interestingly found that E-cadherin was decreased in parous mammary tissue. The data suggest that its regulation may also be dependent on other factors, not limited to YKL-40, such as hormones and their receptors, because E-cadherin expression in breast cancer is associated with increased levels of estrogen receptor [57], [58].

There is a wealth of evidence suggesting that elevated serum levels of YKL-40 in breast cancer patients serve as a cancer prognostic biomarker [29], [59]. However, a compelling study tested and compared YKL-40 levels between the blood and nipple aspirate fluid (NAF) from healthy women and patients with either breast precancer or cancer [60]. Both pre-cancer and cancer patients contained higher concentrations of YKL-40 in NAF than did disease-free women. In addition, YKL-40 in NAF levels from health subjects was 600-fold higher than serum levels of YKL-40 in these normal women or cancer patients. Thus it suggests that NAF levels of YKL-40 may serve as a more sensitive marker than serum levels in the assessment of breast cancer progression. However, substantial epidemiological analyses are essential to firmly establish the relationship between epithelial expression of YKL-40 and pathogenesis of breast cancer from the same patients.

In summary, the data presented here identified that YKL-40 expressed exclusively by ductal epithelia has the ability to inhibit mammary epithelial secretion and differentiation, impair epithelial polarization, and facilitate cell motility in the presence of lactogenic hormones, an essential mechanism that mediates mammary tissue remodeling during involution. These findings underscore the biophysiological activities for YKL-40 and also support the evidence that YKL-40 is significantly elevated in the involution. Therefore, elucidation of its inhibitory activity in mammary epithelial cell differentiation and mammary gland regression will help understand its pathological role in the breast cancer progression that is associated with poor differentiation of cancer cells.

## Materials and Methods

### Culture of 76N MECs

The cells (kindly provided by Drs. Vimla Band and Sallie Smith Schneider) were grown in RPMI 1640 medium with glutamine (Invitrogen) in the presence of 5 µg/ml insulin, 10 µg/ml hEGF, 5 µg/ml hydrocortisone (Sigma, MO, USA), and 10% FBS (Invitrogen).

### Generation of 76N cells stably expressing YKL-40

Full length of YKL-40 cDNA was subcloned into a retroviral pCMV-neo-vector. 293T retroviral packaging cells were transfected with YKL-40 or vector control DNA in the presence of pCL 10A1 vector using Fugene 6 (Roche, IN) as the delivery vehicle. Forty-eight hours after transfection, the supernatant was harvested and filtered through 0.45-µm pore size filters and the viral medium was used to infect 76N MECs. Selection with 800 µg/ml neomycin was started 48 hr after infection and the drug-resistant cell populations were used for subsequent studies.

### Purification of recombinant YKL-40 and polyclonal rAY

Full-length human YKL-40 cDNA with a His-tag was subcloned into a pFastBac1 vector (Invitrogen, CA). Following transformation and amplification in DH10Bac E. coli, bacmid DNA containing YKL-40 was transfected into Sf9 insect cells by using CellFECTIN reagent (Invitrogen) and baculoviral medium was produced. A Ni-NTA column was used to purify recombinant YKL-40 according to manufacture’s instruction (Invitrogen) and YKL-40 pure protein was finally produced through a PD-10 desalting column (Millipore, CA). rAY was purified through an Econo-Pac serum IgG purification kit (BioRad, Hercules, CA) once it was generated from immunization of rabbits with a short peptide of YKL-40 encoding C-terminus of YKL-40.

### 3-D Matrigel assay

76N MECs (2×10^3^) were suspended with 50 µl of assay medium containing 4% growth factor-reduced Matrigel, 5 µg/ml PRL, 5 µg/ml HDC, 5 µg/ml INS and 1% heat-inactivated FBS, and then were transferred onto 96-well plates pre-coated with a layer of Matrigel and incubated for 10-14 days. In some conditions, the cells were suspended with the assay medium in the presence of 100 ng/ml YKL-40 or ySP. Top gel medium was replaced with assay medium every 4 days. To evaluate cell invasive behavior, Matrigel concentration in the assay medium was reduced to 2%.

### Migration assay

76N MECs (2×10^5^P) were transferred onto transwells (8 µm, 24-well plates) pre-coated with collagen IV (100 µg/ml). The lower chamber of transwells included RPMI medium with YKL-40 or ySP. After 4–6 hours of incubation, cells migrated to the membrane were quantified. For migration of 76N MECs expressing YKL-40 or vector, lactogenic hormones (PRL, HDC and INC) were added in the lower chamber.

### Gelatin Zymography

Cell-conditioned serum-free media from 48 hr culture were collected for a zymograph analysis as described previously [57], [58].

### Western Blot Analysis

Cells were collected by scrapping and extracted in a lysis buffer (pH 7.4) containing 0.25 mM HEPES, 14.9 mM NaCl, 10 mM NaF, 2 mM MgCl_2_, 0.5% NP-40, 0.1 mM PMSF, 20 µM pepstatin A and 20 µM leupeptin. The lysates were centrifuged at 10,000 g for 10 min at 4°C and the resulting supernatants were collected for 10% SDS-PAGE. Proteins were transferred to a nylon membrane (Invitrogen) and incubated with one of several antibodies: rabbit anti-YKL-40 (rAY, 1∶200), anti-β-casein (1∶200, Santa Cruz, CA), mouse anti-MMP-9 (1∶500, VWR, NJ), E-cad (1∶500, Invitrogen), and actin (1∶1000, Sigma, MO). Specific bands were detected using an ECL kit (Pierce, VWR).

### Immunofluorescence

Matrigel samples were fixed with 2% para-formaldehyde for 15 min and permeabilized with 0.5% Trition X-100 for 10 min at 4°C. Then the samples were incubated with primary antibodies in PBS-base blocking solution containing 0.1% BSA, 0.2% Triton X-100, 0.05% Tween-20, 7.7 mM NaN_3_, and 10% goat serum overnight at 4°C. Primary antibodies included rabbit anti-milk protein (1∶100; Nordic Immunological Lab, Neitherlands), anti-phospho-Ezrin/Radizin/Moesin (1∶400, Cell Signaling, MA), rat anti-integrin α6 (1∶200, Millipore, MA), and mouse anti-E-cad (1∶500, Invitrogen) and anti-actin (1∶1000, Sigma) antibody. Secondary antibodies including goat anti-mouse or rabbit Alexa Fluor 488 and Alexa Fluor 555 antibody (1∶1000, Invitrogen) were added for 1 hr at room temperature followed by nuclear staining with DAPI (300 nM, Invitrogen).

### RT-PCR

Total RNA was extracted by a Tri-reagent (Molecular Research Inc., OH) from cell lysates. cDNA was synthesized through a reverse transcriptional reaction in the presence of oligo(dT) and a reverse transcriptase (Promega, WI). β-Casein cDNA was amplified through PCR reaction in the presence of 5’ (CAAGGGAGACCATAGAAAGCC) and 3’ (GACACTAATGGGGTTATGAACTGGGGC) DNA primers which covered 600 bp of β-casein gene. Samples (10 µl) were loaded into a 1% agrose gel to determine gene expression levels of β-casein and GAPDH as internal controls.

### 76N MEC transplantation into mammary fat-pad free tissue in mice

All animal experiments were performed under the approval of Institutional Animal Care and Use Committee of the University of Massachusetts (IACUC ID 132669). Transplantation of 76N MECs into cleared fat-pad tissue of 4-week old SCID/Beige mice was described previously [61], [62]. In brief, the 4^th^ nipples and their epithelium-containing parts of the fat pad of mammary glands were excised. The incisions were closed with stitches and seven days later, 76N MECs (6×10^6^) expressing control vector or YKL-40 were injected into the right or left cleared fat-pad tissue, respectively. Host native fat pads and 76N MECs-injected fat pads were removed from 3-month old virgin mice, 7-month old mothers (twenty-one days after giving birth), 10-month old parous mice, and 10-month old non-parous mice.

### Histological analyses

Paraffin-embedded tumor tissues were cut to 6 µm thickness and processed for immunohistochemical analysis. In brief, samples were incubated with 3% H_2_O_2_ for 30 min to block endogenous peroxidase activity, followed by incubation with blocking buffer containing 10% goat serum for 1 hr. The samples then were incubated at room temperature for 2 hr with mouse anti-Ki-67 (1∶100, BD Pharmingen, San Diego, CA), E-cad (1∶2000, Invitrogen), ER (1∶200, Santa Cruz), PR (1∶200, Dako Inc, Carpentaria, CA), and SMa (1∶500, Dako) monoclonal antibodies, or rabbit anti-YKL-40 (1∶200) and CK8 (Abcam, Cambridge, MA) polyclonal antibodies. Goat anti-mouse or anti-rabbit secondary antibodies (1∶100) conjugated to HRP were added for one hr. Finally, DAB substrate (Dako Inc) was introduced for several minutes and after washing, methyl green was used for counterstaining. For detection of tissue apoptosis, we used the TUNEL assay that was described in the instruction of a TACS.XL DAB *in situ* apoptosis detection kit (Trevigen Inc., Gaithersburg, MD).

## Supporting Information

Figure S1 **Pre-incubation of rAY with recombinant YKL-40 blocks rAY binding to tissue-derived YKL-40.** Mammary tissue specimens from parous mice were subjected to IHC using rAY or pre-immune rIgG (1∶200) (the top panel). Recombinant YKL-40 or collagen IV and rAY at 10∶1 molar ratio were pre-incubated overnight at 4°C and then applied to the tissue for IHC analysis of YKL-40 (the bottom panel). Bars: 100 µm.
(JPG)

Figure S2 **YKL-40 does not have effects on proliferation of 76N MECs.** 76N MECs were treated with YKL-40 (100 ng/ml), ySP (100 ng/ml) or PBS in the presence or absence of prolactin (PRL, 5 µg/ml), hydrocortisone (5 µg/ml, HDC), and insulin (5 µg/ml, INS) for 24 hr. 3H-thymide (1 µCi) was introduced to each well for 6 hr. After extensive washes, the cells were scraped and precipitated in 200 µl of 10% TCA and then dissolved in 0.3 ml of 0.3 M NaOH. Radioactivity was quantified by a liquid scintillation counter. n = 5
(JPG)

Figure S3 **Transplanted 76N MECs expressing ectopic YKL-40 develop mammary tissue similar to that derived from control MECs or host inherited cells.** Mammary tissue samples from virgin mice (a-c), mothers at the beginning of weaning (d-f), parous (g-i), and non-parous (j-l) animals were analyzed for IHC of YKL-40. Please note stronger expression of YKL-40 present in epithelia from 76N MECs expressing YKL-40 (c, i, l) than those from control 76N MECs (b, h, k) or host native cells (a, g, j). Bars: 200 µm.
(JPG)
