# Supplementary figures and images for: Inhibitory Activity of YKL-40 in Mammary Epithelial Cell Differentiation and Polarization Induced by Lactogenic Hormones: A Role in Mammary Tissue Involution
Source: PLoS One. 2011 Oct 3;6(10):e25819. doi: 10.1371/journal.pone.0025819 (PMC3185048; doi:10.1371/journal.pone.0025819)

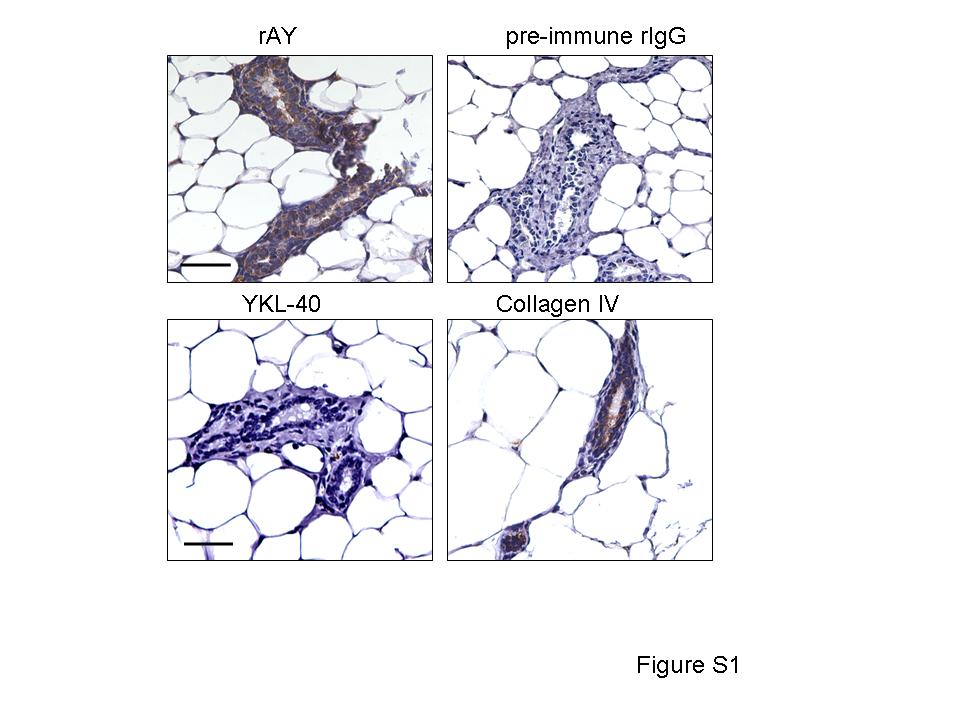

Supplement: Figure S1 — Pre-incubation of rAY with recombinant YKL-40 blocks rAY binding to tissue-derived YKL-40. Mammary tissue specimens from parous mice were subjected to IHC using rAY or pre-immune rIgG (1∶200) (the top panel). Recombinant YKL-40 or collagen IV and rAY at 10∶1 molar ratio were pre-incubated overnight at 4°C and then applied to the tissue for IHC analysis of YKL-40 (the bottom panel). Bars: 100 µm. (JPG) [file pone.0025819.s001.jpg]

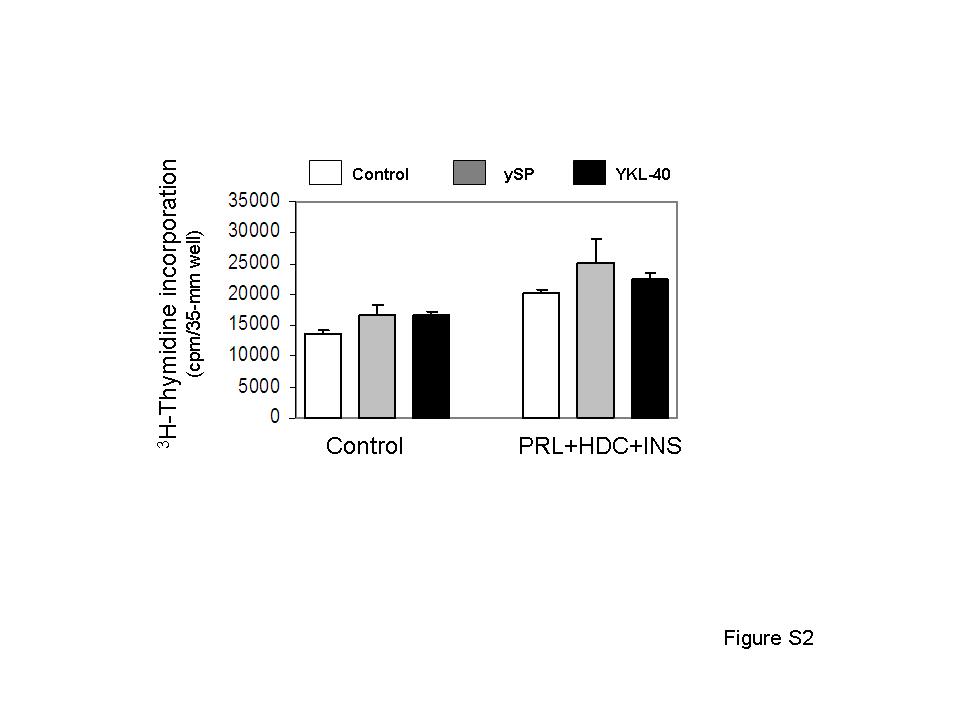

Supplement: Figure S2 — YKL-40 does not have effects on proliferation of 76N MECs. 76N MECs were treated with YKL-40 (100 ng/ml), ySP (100 ng/ml) or PBS in the presence or absence of prolactin (PRL, 5 µg/ml), hydrocortisone (5 µg/ml, HDC), and insulin (5 µg/ml, INS) for 24 hr. 3H-thymide (1 µCi) was introduced to each well for 6 hr. After extensive washes, the cells were scraped and precipitated in 200 µl of 10% TCA and then dissolved in 0.3 ml of 0.3 M NaOH. Radioactivity was quantified by a liquid scintillation counter. n = 5 (JPG) [file pone.0025819.s002.jpg]

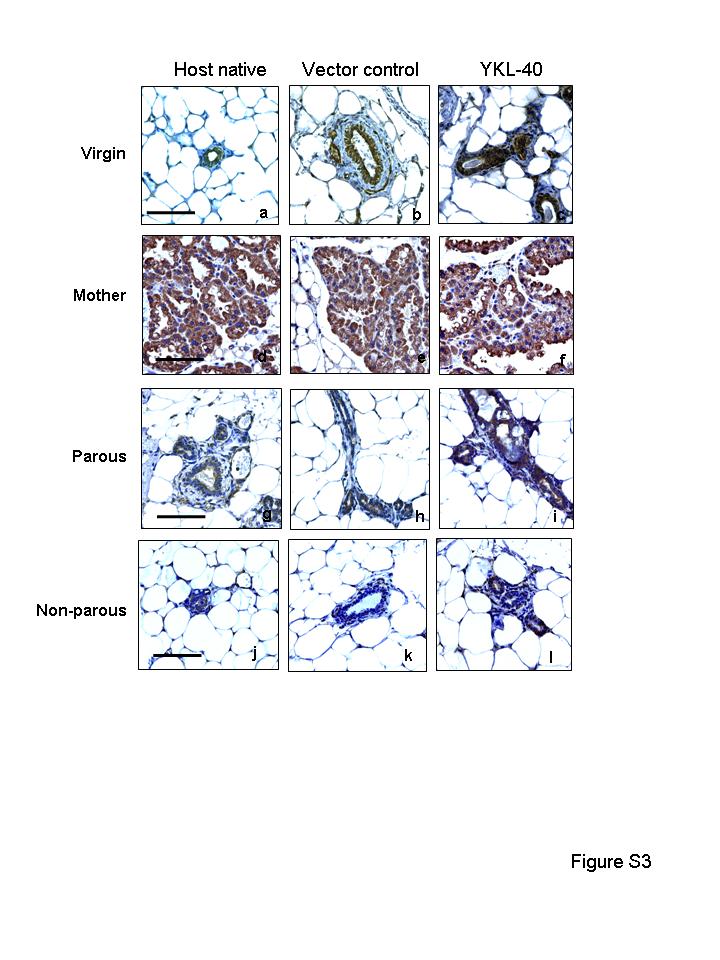

Supplement: Figure S3 — Transplanted 76N MECs expressing ectopic YKL-40 develop mammary tissue similar to that derived from control MECs or host inherited cells. Mammary tissue samples from virgin mice (a-c), mothers at the beginning of weaning (d-f), parous (g-i), and non-parous (j-l) animals were analyzed for IHC of YKL-40. Please note stronger expression of YKL-40 present in epithelia from 76N MECs expressing YKL-40 (c, i, l) than those from control 76N MECs (b, h, k) or host native cells (a, g, j). Bars: 200 µm. (JPG) [file pone.0025819.s003.jpg]
